# Penetrating Obturator Artery Injury after Gunshot Wounds: A Successful Multidisciplinary Trauma Team Approach to a Potentially Lethal Injury

**DOI:** 10.7759/cureus.1857

**Published:** 2017-11-17

**Authors:** Tareq I Maraqa, Ji-sun J Shin, Ismael Diallo, Gul R Sachwani-Daswani, Leo C Mercer

**Affiliations:** 1 Trauma Department, Hurley Medical Center, Michigan State University; 2 Hurley Medical Center, Michigan State University; 3 Trauma and Acute Care Surgery, Hurley Medical Center

**Keywords:** embolization of the obturator artery, obturator artery, internal iliac branches injury, penetrating vascular injury, penetrating pelvic injury, pelvic trauma, penetrating obturator artery injury, gunshot wound

## Abstract

Obturator artery injury (OAI) from pelvic gunshot wounds (GSW) is a rarely reported condition. Hemorrhages from pelvic trauma (PT) are mostly venous. Arterial hemorrhages represent about 10-20% of PTs. When arterial hemorrhages from PT occur, they are a severe and deadly complication often causing significant hemodynamic instability and eventual shock.

*A* 23-year-old male presented to our emergency service via a private vehicle with multiple gunshot wounds to both thighs and to the lower back, resulted in rectal and obturator artery (OA) injuries. The patient underwent a successful coil-embolization of the right OA.

Given the density of structures within the pelvis, patients who sustain gunshot wounds to the pelvic region are at high risk for injury to the small bowel, sigmoid colon, rectum, bladder, and/or vascular structures. While bleeding is the major cause of early mortality in PT, rectal injuries carry the highest mortality due to visceral injuries. A high clinical index of suspicion is needed to diagnose an iliac artery injury or injury to its branches. Prompt computed tomographic angiogram (CTA) and embolization of the OA is the best method to control and stop the bleeding and improve the mortality outcome.

Clinicians caring for patients presenting with pelvic gunshot wounds should pay attention to the delayed presentation of internal hemorrhage from the OAs. A multidisciplinary team approach is crucial in the successful management of penetrating injuries to the obturator artery.

## Introduction

Obturator artery injury (OAI) from pelvic gunshot wounds (P-GSWs) is a rarely reported condition. Though pelvic fractures are the most common cause of hemorrhage in the pelvis, in the setting of pelvic trauma (PT), pelvic gunshot wounds (P-GSWs) may also be a significant cause of pelvic vascular injuries [1-2]. Hemorrhages from PT are mostly venous, with arterial hemorrhages representing about 10-20% of PTs [1]. When arterial hemorrhages from PT occur, they are a severe and deadly complication often causing significant hemodynamic instability and eventual shock [1,3]. Moreover, about 3% of pelvic arterial hemorrhages from gunshot wounds (GSWs) are initially hemodynamically stable and signs of internal bleeding may not be initially evident, delaying the diagnosis [4-5]. We present a case of a 23-year-old male with multiple GSWs to both thighs and to the lower back (sacrum), resulting in OAI requiring arterial embolization.

## Case presentation

A 23-year-old male presented to our emergency service via a private vehicle with multiple GSWs to the upper thighs and sacral area. There was no history of loss of consciousness. During the initial evaluation, the patient’s Glasgow Coma Score (GCS) was 15. His vital signs were as follows: blood pressure 104/84 mmHg, pulse 114 beats per minute, oral temperature 37° celsius, respiratory rate of 26 breaths per minute, and oxygen saturation (SpO2) of 98% on room air.

On physical examination, the patient appeared distressed. Head and neck exams were unremarkable. The lungs were clear to auscultation. A cardiac exam was unremarkable except for tachycardia. Further examination revealed two wounds to the anterior right thigh, one wound to the right lateral thigh, two wounds to the left lateral hip, and one wound to the lower sacral area. There was no active external bleeding upon presentation. Strength and sensation in the lower extremities were intact. Dorsalis pedis and posterior tibialis pulses were 2+ bilaterally. There was no blood on the urethral meatus. A rectal exam revealed fresh blood from the anus with normal sphincter tone. 

**Figure 1:**
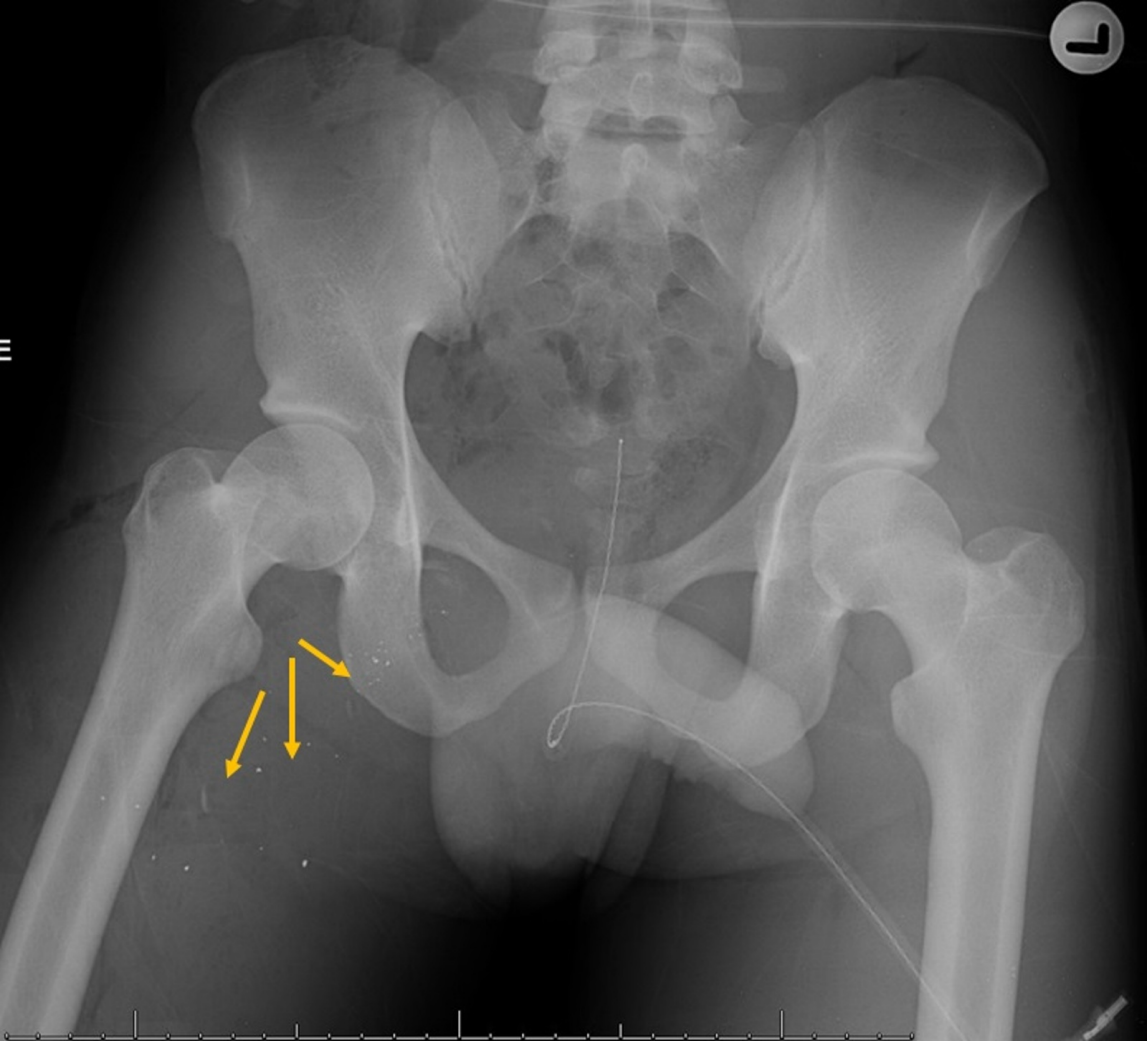
Pelvic X-ray Anterior-posterior (AP) pelvis X-ray demonstrating metallic bullet fragments (arrows) overlying the superior medial right thigh.

A pelvic X-ray revealed dissecting soft tissue emphysema in the proximal right thigh and bullet fragments in the superior medial right thigh with adjacent small calcific fragments without retained large bullet fragments (Figure 1). Fracture of the posterior right obturator ring and fracture of the sacrum were also found. All other radiographs were unremarkable. Because of the fresh anal bleeding, the patient was immediately taken back to the operating room (OR) for exploratory laparotomy due to the concern of an intra-abdominal injury.

Surgical exploration revealed a transmural penetrating injury to the rectum with an associated large hematoma. No gross spillage of rectal content was identified. A diverting end colostomy was subsequently performed. The patient remained sedated and intubated postoperatively and was transferred to the ICU for further monitoring.

**Figure 2:**
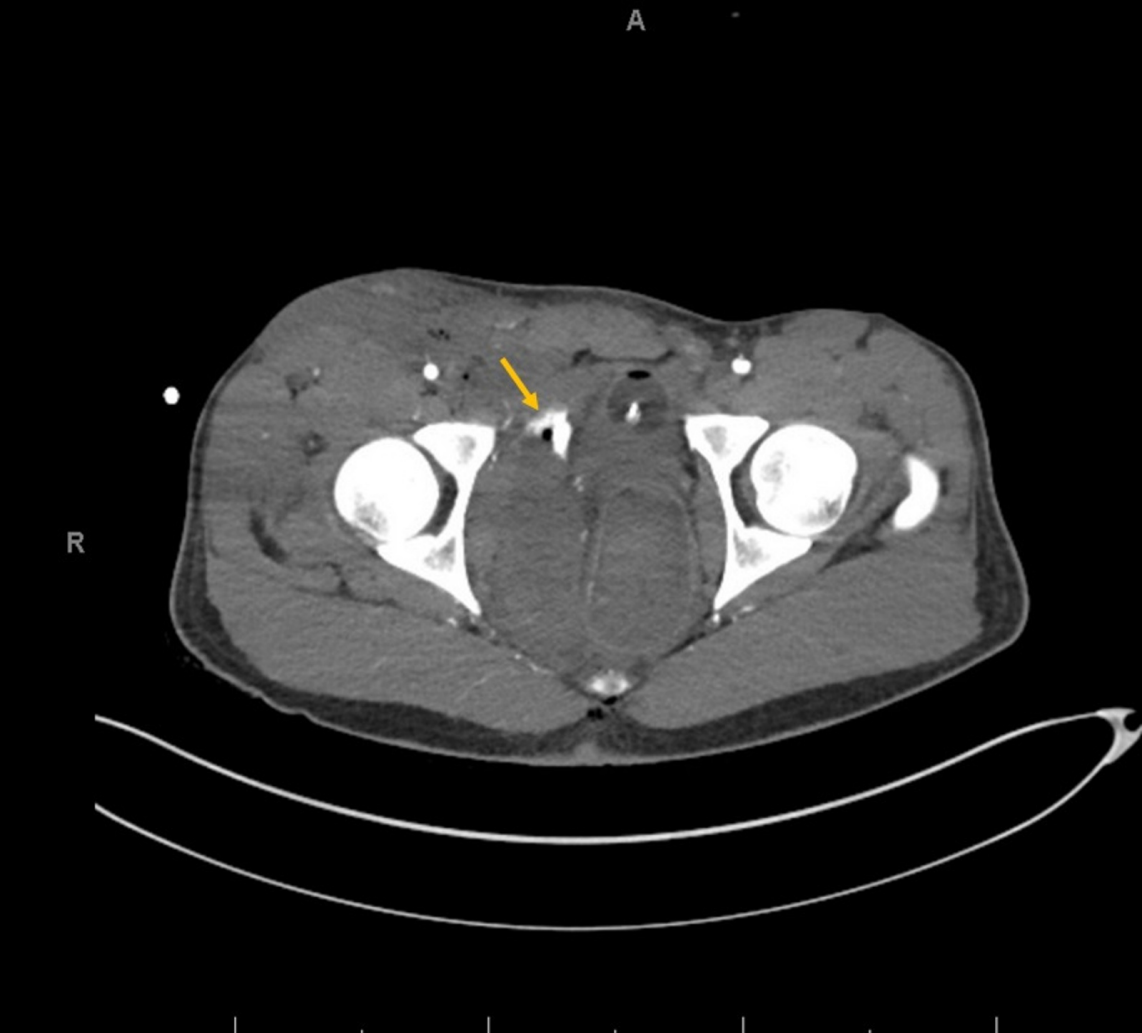
Computed tomography angiography (CTA) axial view Computed tomographic angiography (CTA) axial view of abdominal aorta and bilateral lower extremities demonstrating active extravasation of contrast (arrow) in the right perirectal region extending anteriorly along the right obturator internus displacing the prostate rectal vault and bladder.

**Figure 3:**
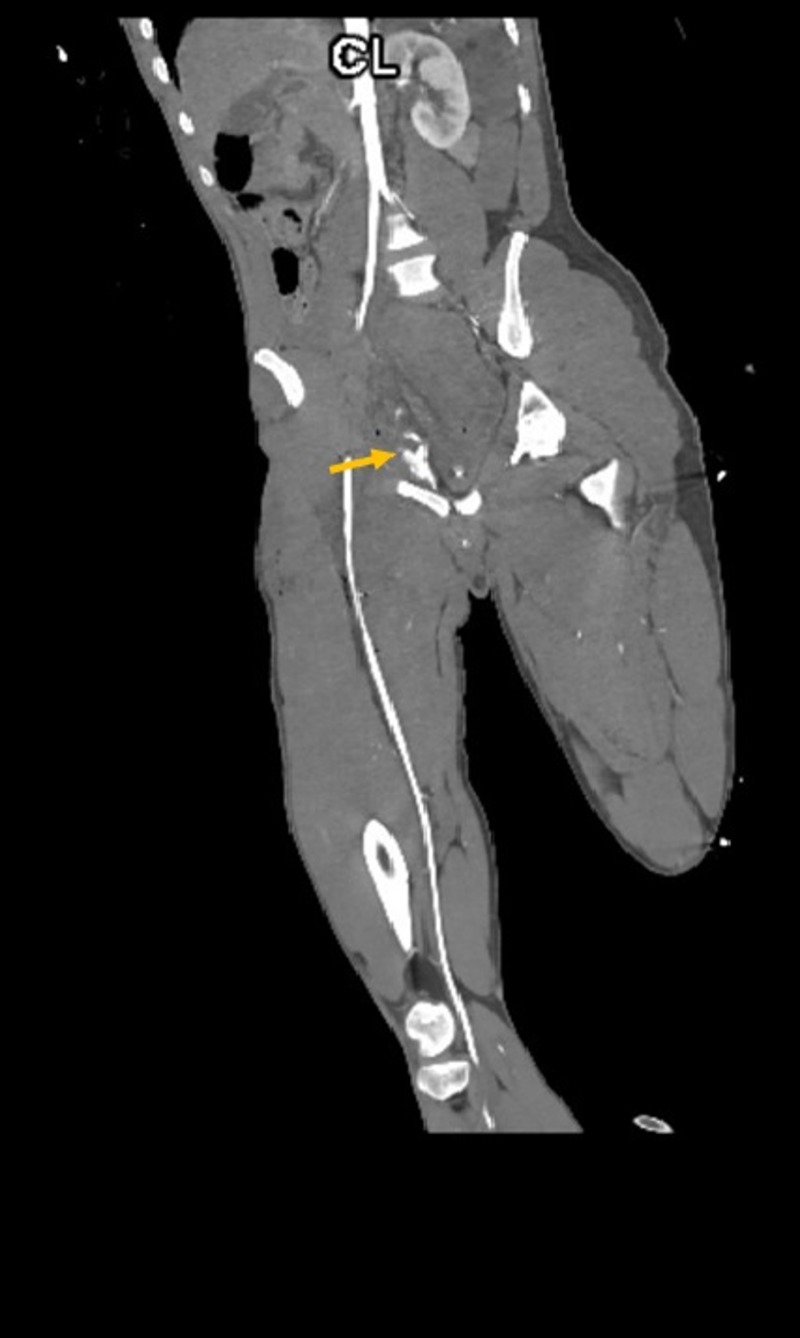
Computed tomography angiography (CTA) of the pelvis (coronal view) Reconstructed coronal view of abdominal aorta and bilateral lower extremities demonstrating blush (arrow) at the superior pubic ramus, consistent with right obturator artery injury.

**Figure 4:**
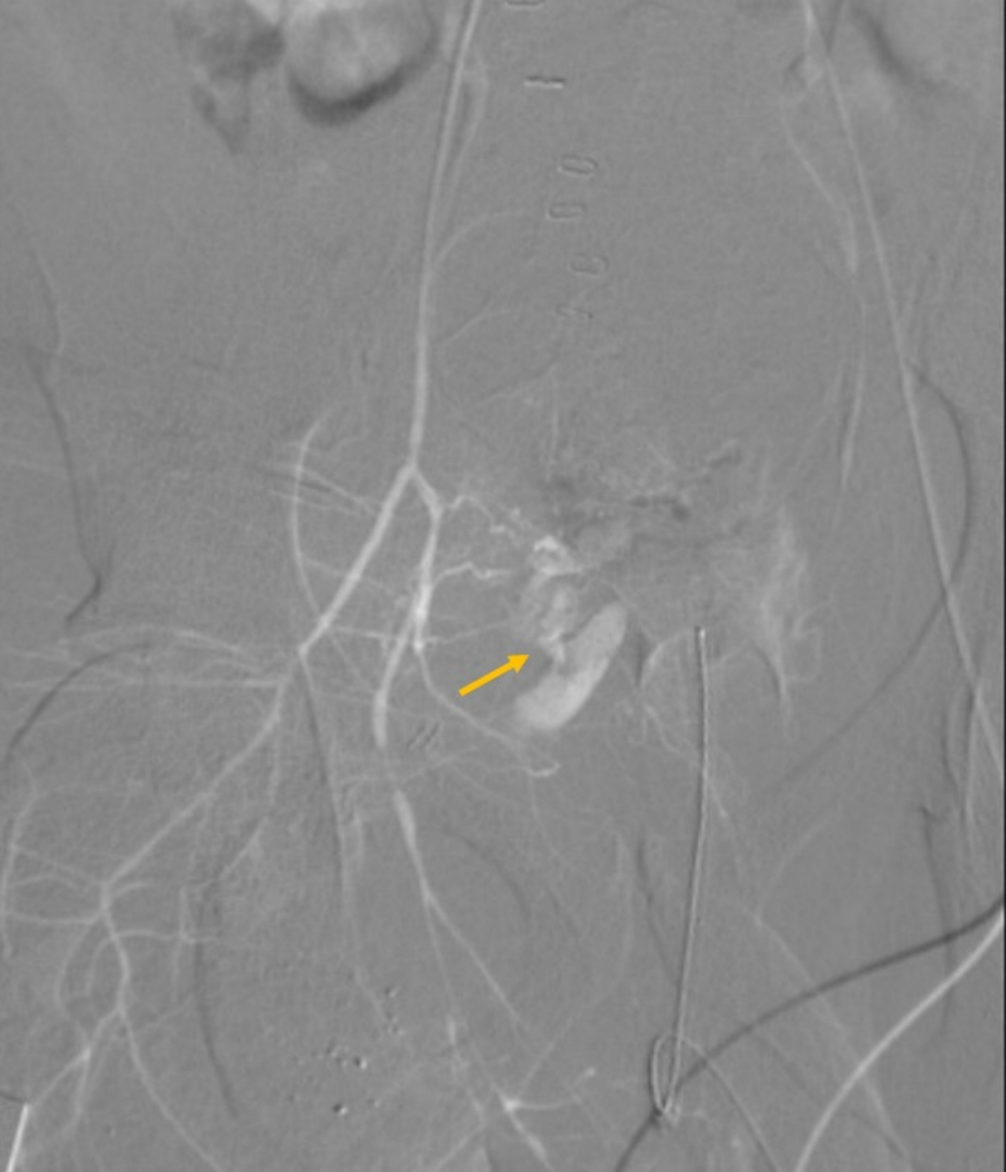
Right iliac angiogram Digital subtraction angiography (DSA) showing active extravasation from region consistent with right obturator artery (arrow).

**Figure 5:**
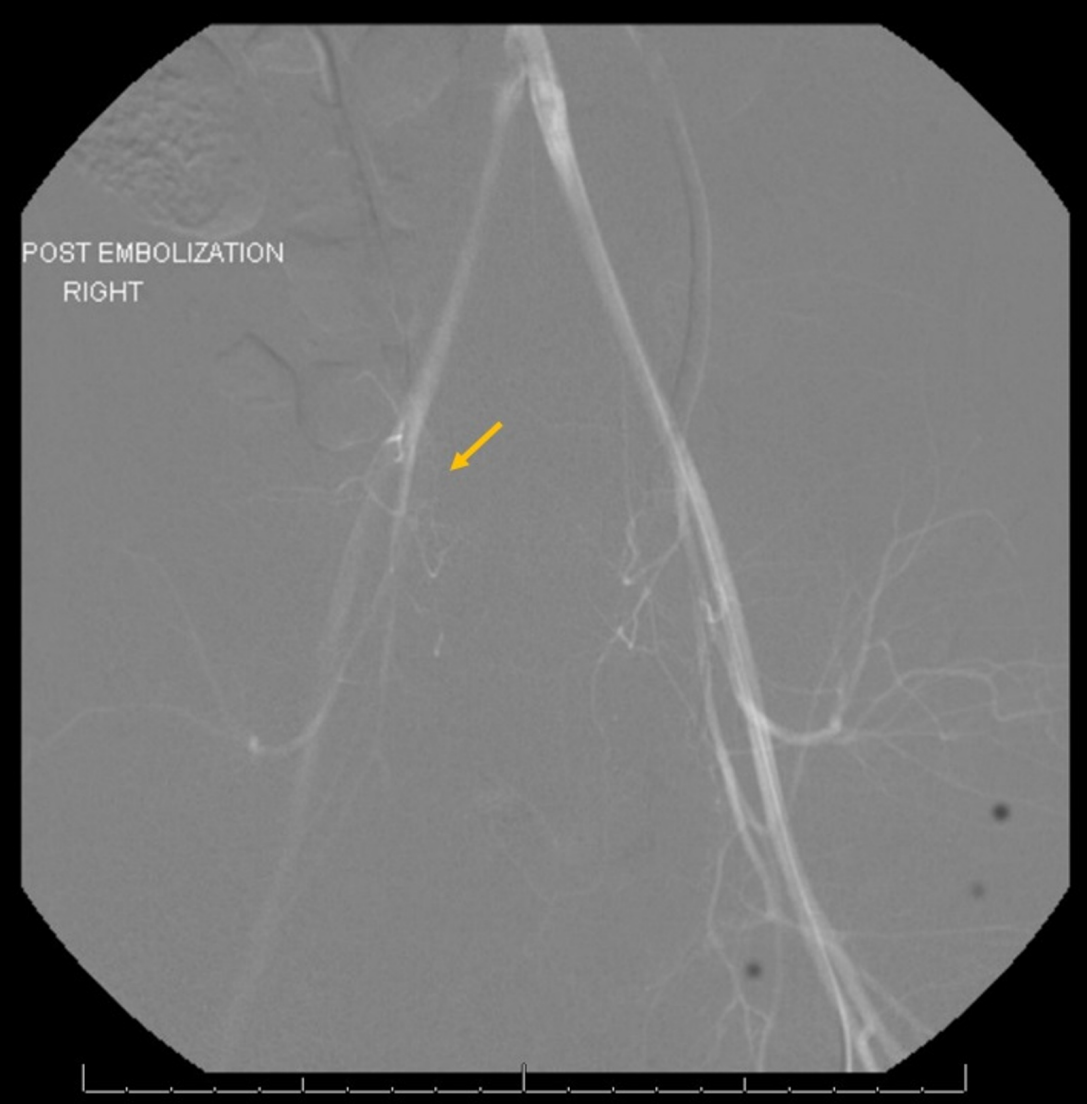
Post-embolization image Post-embolization image showing successful embolization of right obturator artery injury (arrow indicating resolved extravasation from embolized right obturator artery).

Two hours later, the patient became profoundly hypotensive and developed a right groin hematoma. After adequate resuscitation, the patient was taken back to the OR for a right groin exploration. Given the expanding hematoma, a standard longitudinal right groin incision for femoral vessels exposure was performed and a large hematoma was encountered. The hematoma was evacuated. Bleeding was noted to be deep and posterior to the femoral vessels, making surgical control and visualization of the bleeding site extremely challenging. Therefore, the right groin was packed. The patient was sent for a computed tomographic angiogram (CTA), which revealed active extravasation of contrast in the right perirectal region extending anteriorly along the right obturator internus muscle, indicating bleeding from the right OA (Figures 2-3). The patient was taken to interventional radiology (IR) for a coil-embolization of the right OA (Figures 4-5). During the procedure, the patient developed a right pneumothorax, which required needle and tube thoracostomy. Due to a drop in hemoglobin and platelets, the patient received 2 units of packed red blood cells, 10 units of platelets and 5 units of fresh frozen plasma. The patient was stabilized and returned to the ICU for further monitoring.

On postoperative day one (POD-1), the patient was taken back to the OR for right groin wound washout and temporary closure with drain placement. A few hours later, the patient was extubated and was stable in room air. On POD 4, a nasogastric tube was placed due to suspicion of postoperative ileus on radiography. On POD-9, the wound in the patient’s groin started draining copious malodorous brown fluid. A surgical site infection was diagnosed, which resolved with antibiotic treatment and negative-pressure wound therapy. The patient subsequently developed a retroperitoneal abscess, requiring drainage by IR. On POD-20, the patient’s rectal bleeding returned, and he was subsequently taken to the OR for rectal packing. The patient was then taken to IR for angiography, which revealed active bleeding from the left middle rectal artery. The injured artery was successfully embolized.

The remainder of the patient’s hospital course was unremarkable without further complications and he was discharged home in stable condition on POD-30.

## Discussion

About 10% of patients with a traumatic pelvic fracture have concomitant soft tissue injuries and there is a significant increase in mortality, up to 33%, in comparison with patients without concomitant soft tissue injuries [1]. Given the density of structures within the pelvis, patients who sustain GSW to the pelvic region are at high risk for injury to the small bowel, sigmoid colon, rectum, bladder, and/or vascular structures [5]. Although bleeding is the major cause of early mortality, due to the rich vascular supply and the difficulty in achieving surgical control of pelvic hemorrhage, rectal injuries carry the highest mortality due to pelvic visceral injuries [6-7]. Our patient had fractures to the sacrum and obturator ring in addition to rectal and obturator artery injuries. Reviewing the computed tomography (CT) scan and angiogram with the radiologist revealed that the obturator artery injury occurred due to the direct effect of the bullet. Furthermore, the most likely reason for the hematochezia was the blood channeled through the rectal injury. Moreover, this would explain why there was no massive blood collection in the pelvis.

The OA originates from the anterior trunk of the internal iliac artery in the majority of the population. However, aberrant OAs known as the corona mortis are not uncommon. The corona mortis is an anastomosis between the OA and the inferior epigastric artery, which is a branch of the external iliac artery [3]. About 55% of analyzed pelvises on CTA have an incidence of aberrant OA, which is often a source of unidentified and difficult-to-control hemorrhage in PT patients [3-4]. A high index of suspicion is needed to diagnose an iliac artery injury or injury to its branches. Certain signs include the following: persistent hypotension refractory to fluid resuscitation, pelvic fractures, multiple pelvic trauma sites, and expanding groin hematomas [8]. Prompt CTA followed by embolization of the OA is the best method to control and stop the bleeding and improve the mortality outcome [1,7]. Patients who are hypotensive or require blood transfusion may need re-embolization [7].

Embolization of the pelvic vessels is considered safe because of the extensive collateralization [7]. However, embolization of the internal iliac artery may be associated with major ischemic complications such as gluteal and bladder necrosis, surgical wound breakdown, paresthesia, deep infections, and impotence [7,9]. Bilateral embolization of the pelvic vessels is associated with greater complications compared to unilateral embolization, [9] similar to our patient. Our patient had selective unilateral embolization of the OA, which is associated with a good outcome. In addition, our patient had left middle rectal artery embolization without any further complications. The true complication rate of embolization of pelvic vessels in trauma patients is hard to asses, as trauma itself can cause complications [7]. Our patient developed a deep pelvic abscess, which could be a complication of the arterial embolization, penetrating pelvic injury, or the rectal injury. Indications for arterial embolization are hemodynamically unstable patients, who have contrast extravasation seen on CTA or patients older than 60 years and have a major pelvic fracture [7]. Blood transfusions in PT patients with fracture and hemorrhage are common and the median 24 hour red blood cells (RBCs) transfusion is 5 units, and about 30% of patients require massive transfusion of at least 10 units of RBCs [10]. Our patient received a total of 2 units of RBCs, which is significantly below this median.

## Conclusions

Clinicians caring for patients presenting with pelvic GSWs should pay attention to the delayed presentation of internal hemorrhage from the OAs. The best method of diagnosis and management is CTA and embolization, respectively, with close attention to the presence of an aberrant OA. A multidisciplinary team approach is crucial in the successful management of penetrating injuries to the obturator artery.
